# THE MODERATING EFFECT OF AGE ON THE ASSOCIATION BETWEEN ANTICIPATORY APPRAISALS AND AFFECTIVE REACTIVITY

**DOI:** 10.1093/geroni/igae098.2709

**Published:** 2024-12-31

**Authors:** Lana Kinnear, Gloria Luong

**Affiliations:** Colorado State University, Fort Collins, Colorado, United States; Colorado State University, Fort Collins, Colorado, United States

## Abstract

Affective reactivity (changes in emotion in response to a stressor) has been linked to poorer health. Variability in affective reactivity may be due to individual differences in anticipatory appraisals (AAs; perceptions of threat associated with an upcoming stressor). More work is needed to understand how AA predicts affective reactivity to stressors, and adult age differences in these links. Theoretical frameworks suggest older adults may appraise upcoming stressors as more positive compared to younger adults, but it is unclear if anticipatory appraisals reduce affective reactivity differently across age groups. This research leveraged data from 159 younger and older adult participants in the Health and Daily Experiences (HEADE) study to examine how AA predicts affective reactivity to a well validated lab stressor (Trier Social Stress Test) involving spontaneous public speaking and mental arithmetic tasks. Participants completed ecological momentary assessments which included phone surveys assessing anticipatory appraisals of the upcoming lab session. It was hypothesized that more positive AAs would predict lower positive affective (PA) and negative affective (NA) reactivity, and that this effect will be more pronounced for older, relative to younger, adults. Multiple regression analyses revealed that contrary to the hypothesis, more positive AAs were associated with an overall increase in PA, but not NA, reactivity among older adults. This effect was not found for younger adults. These findings emphasize the importance of understanding how overly positive anticipatory appraisals may exacerbate stress responses in older adulthood.

